# AtSWEET13 and AtSWEET14 regulate gibberellin-mediated physiological processes

**DOI:** 10.1038/ncomms13245

**Published:** 2016-10-26

**Authors:** Yuri Kanno, Takaya Oikawa, Yasutaka Chiba, Yasuhiro Ishimaru, Takafumi Shimizu, Naoto Sano, Tomokazu Koshiba, Yuji Kamiya, Minoru Ueda, Mitsunori Seo

**Affiliations:** 1 RIKEN Center for Sustainable Resource Science, 1-7-22 Suehiro-cho, Tsurumi-ku, Yokohama, Kanagawa 230-0045, Japan; 2 Graduate School of Science, Tohoku University, 6-3, Aramaki-Aza-Aoba, Aoba-ku, Sendai 980-8578, Japan; 3 Department of Biological Sciences, Tokyo Metropolitan University, 1-1 Minami-Osawa, Hachioji, Tokyo 192-0397, Japan

## Abstract

Transmembrane transport of plant hormones is required for plant growth and development. Despite reports of a number of proteins that can transport the plant hormone gibberellin (GA), the mechanistic basis for GA transport and the identities of the transporters involved remain incomplete. Here, we provide evidence that *Arabidopsis* SWEET proteins, AtSWEET13 and AtSWEET14, which are members of a family that had previously been linked to sugar transport, are able to mediate cellular GA uptake when expressed in yeast and oocytes. A double *sweet13 sweet14* mutant has a defect in anther dehiscence and this phenotype can be reversed by exogenous GA treatment. In addition, *sweet13 sweet14* exhibits altered long distant transport of exogenously applied GA and altered responses to GA during germination and seedling stages. These results suggest that AtSWEET13 and AtSWEET14 may be involved in modulating GA response in *Arabidopsis*.

## Abstract (web summary)

SWEET proteins are known to function as sugar transporters. Here, Kanno *et al*. show that *Arabidopsis* SWEET13 and SWEET14 are also able to transport the plant hormone gibberellin (GA) in heterologous systems and that *sweet* mutants display phenotypes consistent with altered GA response.

Transport of solutes across non-permeable biological membranes consisting of a lipid bilayer is required for many aspects of biological processes, including generation of electrochemical potentials, energy production, metabolism, signal transduction and so on, and membrane transporters are the proteins that mediate these processes. Plants produce a variety of compounds and metabolites, which accumulate in specific tissues, cell types and subcellular compartments via transport^1^, indicating the presence of highly sophisticated transport mechanisms in plants.

Plant hormones are a group of small molecules that induce a wide range of physiological responses at low concentrations (less than μM if applied exogenously). The transport of auxin (indole-3-acetic acid; IAA) is well known to be required for many physiological processes during plant growth and development^2^,^3^. Plant-specific PIN-FORMED (PIN) transporters mediate IAA efflux from cells, whereas the amino acid permease-like AUX/LUX family proteins import IAA into cells. In addition, some members in subgroup B of the ATP-binding cassette (ABC)-type transporter family (ABCB) function as IAA transporters. Involvement of ABC transporters in the transport of other plant hormones has also been reported. In *Arabidopsis*, AtABCG25 functions as an abscisic acid (ABA) transporter that mediates ABA export from ABA biosynthesizing cells around vascular tissues^4^,^5^, whereas AtABCG40 is an ABA importer involved in the uptake of ABA into guard cells to regulate stomatal aperture^6^. Furthermore, in *Arabidopsis*, AtABCG14 regulates root-to-shoot transport of the cytokinin *trans*-zeatin, to modulate shoot development^7^,^8^. In petunia, the ABCG protein PDR1 regulates symbiotic interactions with arbuscular mycorrhizae and shoot branching by regulating cellular strigolactone export^9^.

Recently, some of the *Arabidopsis* NRT1/PTR FAMILY (NPF) proteins, initially characterized as nitrate or di/tri-peptide transporters^10^,^11^, were also identified as plant hormone transporters. AtNPF6.3 was originally identified as a dual affinity nitrate transporter CHL1/NRT1.1 that is regulated by protein phosphorylation^12^,^13^; however, AtNPF6.3/CHL1/NRT1.1 reportedly transports IAA with competition between nitrate and IAA playing an important role in lateral root development^14^. In our previous study, we identified the low affinity nitrate transporter NPF4.6/NRT1.2 (ref. 15) as a protein capable of transporting ABA by a modified yeast two-hybrid (Y2H) screening system using the ABA receptor complex as a sensor to detect ABA concentrations in yeast cells^16^. Mutants defective in AtNPF4.6/NRT1.2 had more open stomata compared with the wild type, indicating that the protein functions as an ABA transporter *in vivo*. We subsequently identified additional *Arabidopsis* NPF proteins capable of transporting gibberellin (GA) and jasmonoyl-isoleucine (JA-Ile) as well as ABA, although the functions of these proteins *in vivo* are unknown^17^. Independently, *AtNPF2.10/GTR1* was identified as a gene that is co-expressed with jasmonate biosynthesis genes^18^. AtNPF2.10/GTR1 had been characterized as a glucosinolate transporter^19^; however, AtNPF2.10/GTR transports not only glucosinolate but also GA and JA-Ile when expressed in *Xenopus* oocytes. Furthermore, the *gtr1* mutant is less sensitive to exogenously applied methyl jasmonate in seedlings, and the reduced fertility observed in *gtr1* was restored by GA application, suggesting that this protein can function as a GA/JA-Ile transporter^18^. AtNPF3.1 was reported recently to function as a GA transporter *in vivo*^20^. In addition to the above-mentioned transporters, the multidrug and toxin extrusion (MATE) family of proteins has been implicated in salicylic acid (SA)^21^ and ABA^22^ transport. The existence of multiple plant hormone transporters and transporter families for a particular plant hormone suggests that plants have highly redundant hormone transport systems.

GA regulates diverse aspects of plant growth and development, including seed germination, stem elongation, leaf expansion and flower and seed development^23^. As mentioned above, *Arabidopsis* AtNPF2.10/GTR1 and AtNPF3.1 have been identified as GA transporters^18^,^20^; however, since the GA-related phenotypes of the mutants defective in AtNPF2.10/GTR1 or AtNPF3.1 are limited compared with those observed in severe GA-deficient or signalling mutants^24^,^25^, there might be other GA transporters that have not yet been identified. In this work, we conducted a functional screen for GA transporters using a Y2H system with the GA receptor GID1a and the DELLA protein GAI, and found that some *Arabidopsis* SWEET proteins transport GA when expressed in yeast and *Xenopus* oocytes. SWEET proteins were originally identified as a novel class of sugar transporters that facilitate transmembrane transport of sugars like glucose, fructose and/or sucrose bi-directionally depending on their concentrations^26^. In *Arabidopsis*, AtSWEET11 and AtSWEET12 are localized to the plasma membrane of phloem parenchyma cells and mediate phloem loading of sucrose^27^. AtSWEET11 and AtSWEET12 are also expressed in developing seeds together with AtSWEET15, and they regulate sucrose transport from the seed coat to the embryo through the endosperm^28^. In contrast, AtSWEET2, AtSWEET16 and AtSWEET17 mediate vacuolar sugar transport^29^,^30^,^31^,^32^. AtSWEET17 regulates the fructose content in leaf vacuoles^29^, whereas AtSWEET2 modulates sugar secretion from roots and is involved in resistance to the pathogen *Pythium*^32^. These diverse functions suggest that SWEET proteins play important roles in various physiological processes. In the present study we show that AtSWEET13 and AtSWEET14 are required for proper development of anthers, seeds and seedlings. Our data suggest that the functions of AtSWEET13 and AtSWEET14 are associated with GA-mediated physiological responses.

## Results

### Screen for potential GA transporters

To identify novel GA transporters, we screened for cDNAs that induced interactions between the *Arabidopsis* GA receptor GID1a and the DELLA protein GAI at a low GA concentration (0.1 μM) using a Y2H system^17^. GA_3_, the major bioactive GA produced in the fungus *Gibberella fujikuroi*, was used as a substrate because it is commercially available in relatively large quantities. Furthermore, GA_3_ is resistant to hydroxylation at the C-2 position due to the presence of a double bond between C-2 and C-3, and is therefore predicted to have stable biological activity. We transformed ∼1.9 × 10^6^ cDNAs cloned in a yeast expression vector, which originated from ∼0.5 × 10^6^ initial independent cDNAs that were synthesized from RNA extracted from 2-week-old *Arabidopsis* seedlings, into yeast cells containing the BD-GID1a and AD-GAI constructs. In the first screen, we obtained 131 colonies that grew on selection media containing 0.1 μM GA_3_ using *HIS3* as a positive selection marker. In the second screen, 11 clones that grew on selection media in the presence, but not in the absence, of 0.1 μM GA_3_ were selected. Among the 11 clones, three contained cDNAs corresponding to *AtNPF1.2* (At1g52190), and another three clones contained cDNAs corresponding to *AtSWEET13* (At5g50800). AtNPF1.2 has been reported already to transport GA^17^, which indicated that the screening system performed as expected. We hypothesized that AtSWEET13, which belongs to the recently identified sugar transporter SWEET family^26^,^27^,^33^, could transport multiple substrates as has been shown for *Arabidopsis* NPF proteins^17^.

**Figure 1:**
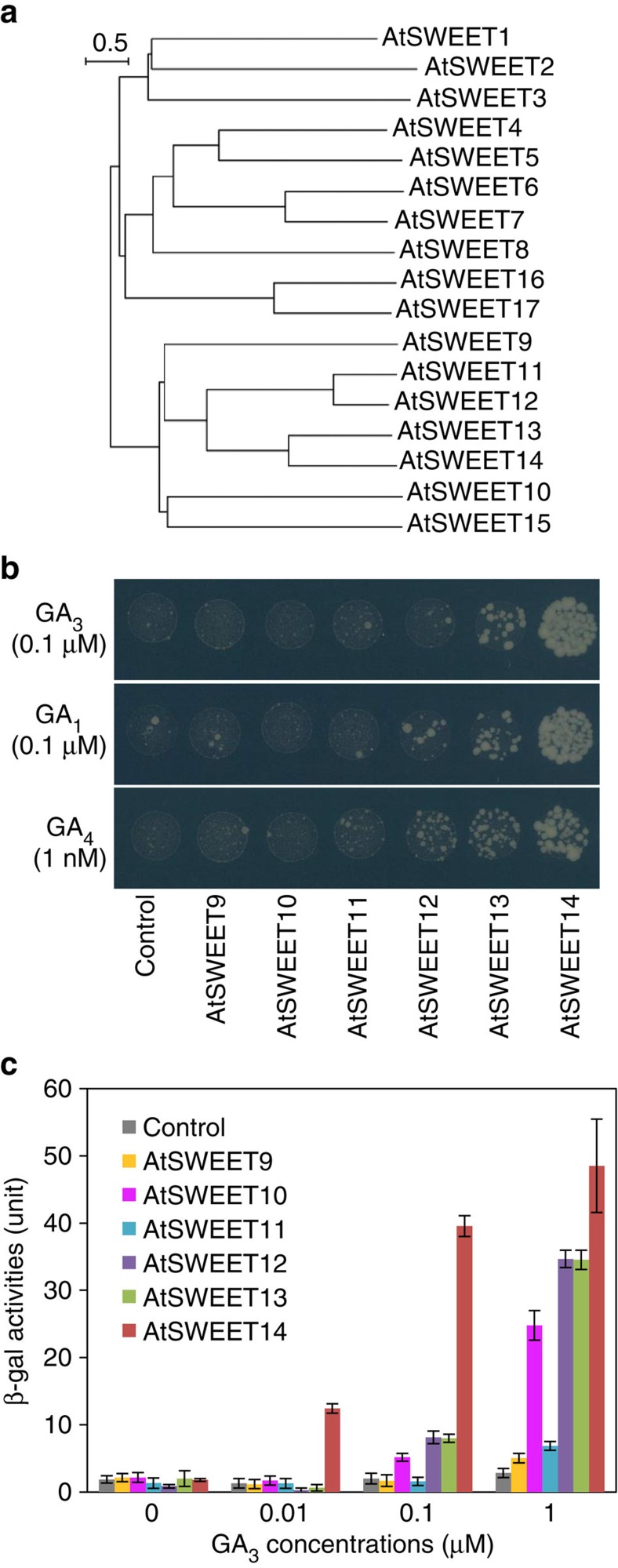
Identification of *Arabidopsis* SWEET proteins as candidate GA transporters. (**a**) A phylogenetic tree of 17 *Arabidopsis* SWEET proteins was generated by ClustalX using the neighbour-joining method and displayed using Njplot. The scale bar indicates a branch length corresponding to 0.5 substitutions per site. (**b**) The effects of six closely related AtSWEET proteins on the interaction between GID1a and GAI determined by Y2H assays using *HIS3* as a selection marker. AtSWEET9, AtSWEET10, AtSWEET11, AtSWEET12, AtSWEET13 or AtSWEET14 was expressed in yeast containing the Y2H system with BD-GID1a and AD-GAI, and grown on selection media (SD; -Leu, -Trp, -Ura, -His) containing GA_1_ (0.1 μM), GA_3_ (0.1 μM) or GA_4_ (1 nM) for 7 days. Yeast transformed with an empty vector was used as a control. 1 × 10^4^ cells were inoculated on the media. (**c**) The effects of six closely related AtSWEET proteins on the interaction between GID1a and GAI were determined by Y2H assays based on the expression of the *lacZ* marker. Units of β-galactosidase (β-gal) activity represent 1,000 × OD_420_/min/OD_600_ cells. Values are means±s.d. of three biological replicates.

Next, we examined whether some other proteins related to AtSWEET13 would be able to induce BD-GID1a/AD-GAI interactions in yeast (Fig. 1). We cloned five additional cDNAs encoding AtSWEET9, 10, 11, 12 and 14 (Fig. 1a), and expressed the proteins in yeast containing the Y2H BD-GID1a/AD-GAI system. In the presence of 0.1 μM GA_3_, AtSWEET14 enhanced yeast growth on selection media more effectively than did AtSWEET13 (Fig. 1b). Similar results were obtained when GA_1_ (0.1 μM) or GA_4_ (1 nM) was used as a substrate. Under these conditions, the effects of other AtSWEET proteins on yeast growth were not clear. Therefore, we examined the BD-GID1a/AD-GAI interactions using a more sensitive *lacZ* marker for a β-galactosidase (β-gal) assay (Fig. 1c). As predicted from yeast growth on selection media, AtSWEET14 promoted BD-GID1a/AD-GAI interactions more efficiently than did the other AtSWEETs, and 10 nM GA_3_ was sufficient to induce detectable β-gal activity. In addition, AtSWEET10 and AtSWEET12 enhanced the BD-GID1a/AD-GAI interactions to levels similar to AtSWEET13 but to a lesser extent than AtSWEET14.

### GA transport activities of AtSWEET proteins

The close phylogenetic relationship as well as similar gene expression patterns predicted by a public database (*Arabidopsis* eFP Browser; http://bar.utoronto.ca/efp/cgi-bin/efpWeb.cgi; Supplementary Fig. 1)^34^,^35^,^36^ suggested that AtSWEET13 and AtSWEET14 have redundant physiological roles. AtSWEET13 and AtSWEET14 have been shown to transport sucrose when expressed in human embryonic kidney (HEK) 293T cells^27^. AtSWEET13 has also been reported to regulate pollen wall pattern formation in association with AtSWEET8 (ref. 37); however, the *in vivo* functions of AtSWEET13 and AtSWEET14 remain largely unknown.

**Figure 2:**
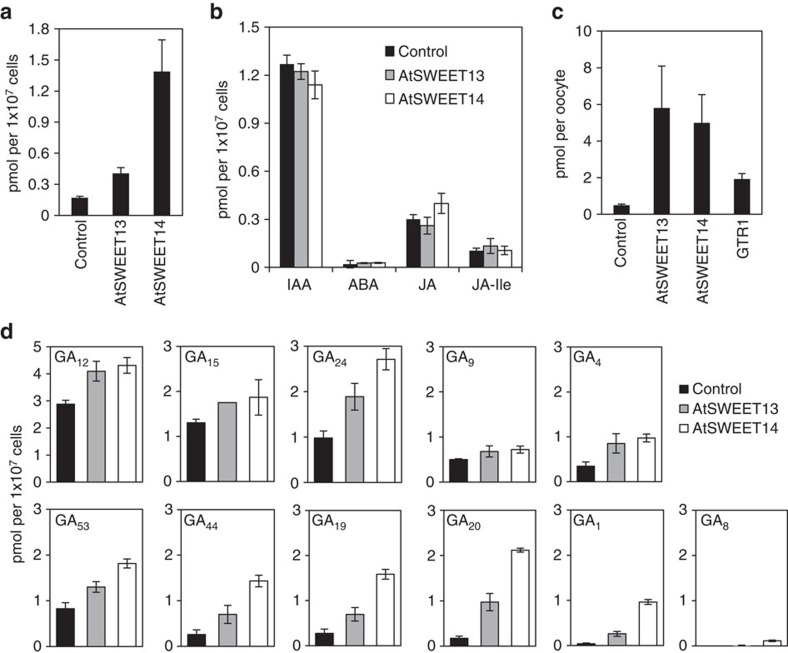
AtSWEET13 and AtSWEET14 transport GA. (**a**) GA transport activities of AtSWEET13 and AtSWEET14 in yeast. Yeast cells expressing AtSWEET13 or AtSWEET14 were incubated with 10 μM GA_3_ in the presence 100 mM glucose at pH 5.8. Yeast transformed with an empty vector was used as a control. (**b**) Plant hormone transport activity of AtSWET13 and AtSWEET14. Yeast cells expressing AtSWEET13 or AtSWEET14 were incubated with 10 μM IAA, ABA, JA or JA-Ile in the presence 100 mM glucose at pH 5.8. Yeast transformed with an empty vector was used as a control. (**c**) GA transport activities of AtSWEET13 and AtSWEET14 in *Xenopus* oocytes. Oocytes injected with *AtSWEET13* or *AtSWEET14* cRNA were incubated for 24 h in Kulori medium-based buffer (pH 5.0) containing 100 μM GA_3_. As a control, water was injected into the oocytes. *AtNPF2.10*/*GTR1* cRNA was also injected for comparison. Values are means±s.d. of four biological replicates with two oocytes. (**d**) Substrate preferences of AtSWEET13 and AtSWEET14 for different GA species. Yeast cells expressing AtSWEET13 or AtSWEET14 were incubated with a mixture of 11 GAs (5 μM each) in the presence of 100 mM glucose at pH 5.8. Yeast transformed with an empty vector was used as a control. For (**a**,**b**,**d**), compounds taken into cells after incubation for 5 min were analysed by LC–MS/MS. Values are means±s.d. of three biological replicates.

To assess GA transport activity more directly, we quantified GA taken into yeast cells or *Xenopus* oocytes by mass spectrometry (Fig. 2). In the buffer conditions used previously for AtNPFs (50 mM potassium phosphate buffer, pH 5.8–7.0)^16^,^17^,^38^, we were unable to detect significant GA transport activities by AtSWEET13 and AtSWEET14 in yeast (Supplementary Fig. 2a). As shown in Fig. 1, AtSWEET13 and AtSWEET14 induced BD-GID1a/AD-GAI interactions in yeast when the cells were incubated in growth media. Thus, we suspected that elements present in the media were required for these activities. We found that a significant amount of GA_3_ was taken into yeast expressing AtSWEET13 or AtSWEET14 when 100 mM glucose was added to the reaction buffer at acidic pH conditions (Fig. 2a; Supplementary Fig. 2a). This phenomenon appeared not to be direct regulation of AtSWEET13 and AtSWEET14 by glucose because similar induction of GA transport activity was observed for AtNPF2.5 that was previously shown to induce BD-GID1a/AD-GAI interactions in yeast^17^ (Supplementary Fig. 2b). In the same conditions, yeast cells expressing AtSWEET13 or AtSWEET14 accumulated ABA, IAA, jasmonic acid (JA) and JA-Ile at levels similar to control cells that did not possess the transporters (Fig. 2b), suggesting that the proteins mediated specific GA transport. We further confirmed the GA transport activities of AtSWEET13 and AtSWEET14 using *Xenopus* oocytes. Oocytes injected with either *AtSWEET13* or *AtSWEET14* cRNA accumulated significantly higher levels of GA_3_ than water-injected control oocytes, and the levels were higher even if compared with oocytes injected with previously characterized *AtNPF2.10*/*GTR1* cRNA (Fig. 2c). The activities were detected even in the absence of glucose (Kulori medium-based buffer) and were not affected by the addition of glucose (Supplementary Fig. 2c). Again, this finding suggests that glucose does not directly regulate the GA transport activities of AtSWEET13 and AtSWEET14. In yeast, GA_3_ uptake into cells mediated by AtSWEET14 was saturated rapidly between 2.5 and 5 min (Supplementary Fig. 2d), and it was technically difficult to determine the uptake kinetics in this system. In contrast, GA_3_ uptake into *Xenopus* oocytes mediated by AtSWEET13 and AtSWEET14 was more gradual (Supplementary Fig. 2e). In this system, the *K*m values of both AtSWEET13 and SWEET14 for GA_3_ were estimated to be several hundred μM (Supplementary Fig. 2f,g).

Next, we tested whether AtSWEET13 and AtSWEET14 could transport different types of GAs using the yeast assay system (Fig. 2d). We used a mixture of 11 GAs (5 μM each), including two major bioactive GAs in *Arabidopsis*, GA_1_ and GA_4_, as well as their precursors and metabolites, for which isotope-labelled internal standards were available, as substrates. Most of the GAs accumulated at higher levels in yeast expressing AtSWEET13 or AtSWEET14, compared with yeast that were not expressing the transporters, indicating that these proteins transport various GAs; however, each of the 11 GAs appeared to have a different membrane permeability. These differences in membrane permeability are possibly due to their different hydrophobicities; GA_12_ uptake was high in yeast cells even in the absence of transporters, and the permeability was gradually lower after successive oxidations of GA_12_ by GA13ox, GA20ox, GA3ox and GA2ox (Fig. 2d; Supplementary Fig. 2h). This result suggests that specific transporters are required for GAs that have a lower membrane permeability, such as GA_1_.

### Spatial expression patterns of *AtSWEET13* and *AtSWEET14*

**Figure 3:**
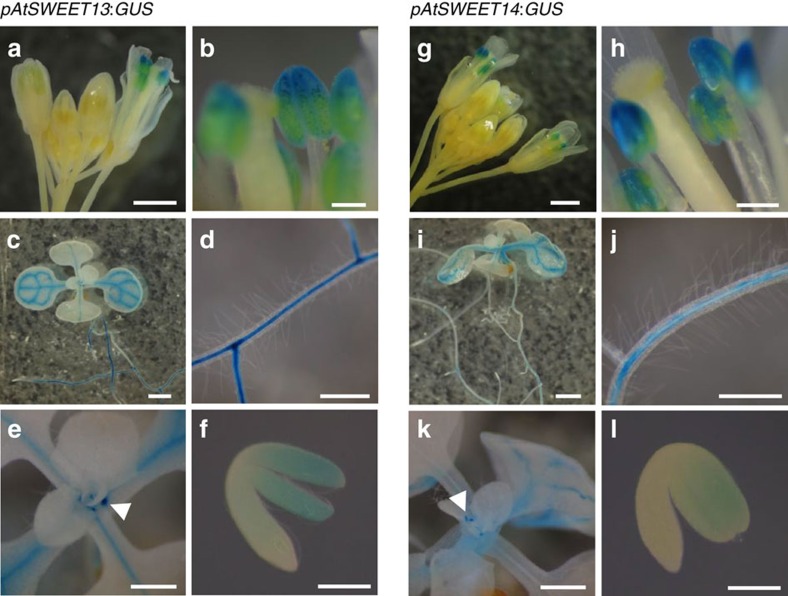
Spatial expression patterns of *AtSWEET13* and *AtSWEET14.* (**a**–**f**) GUS activities of *pAtSWEET13:GUS* transgenic plants. (**g**–**l**) GUS activities of *pAtSWEET14:GUS* transgenic plants. (**a**,**g**), flower buds; (**b**,**h**), anthers; (**c**,**i**), shoots of 2-week-old seedlings; (**d**,**j**), roots of 2-week-old seedlings; (**e**,**k**), junctions of stems and petioles of 2-week-old seedlings; (**f**,**l**) embryos at 15 days after flowering. Arrowheads in **e** and **k** show dot-like staining at the junctions of stems and petioles. Scale bars: 1 mm for **a**,**c**,**g** and **i**, 0.2 mm for **b**,**f**,**h** and **l**, 0.5 mm for **d**,**e**,**j** and **k**.

To investigate the spatial expression patterns of *AtSWEET13* and *AtSWEET14*, we generated transgenic plants expressing the *GUS* gene under the control of the *AtSWEET13* and *AtSWEET14* promoters (*pAtSWEET13:GUS* and *pAtSWEET14:GUS*, respectively) (Fig. 3). As predicted by a public database (Supplementary Fig. 1), the promoter activities of *AtSWEET13* and *AtSWEET14* were detected in stamens during the later stages of flower development (Fig. 3a,g). Closer investigation revealed that this expression was localized to the anthers (Fig. 3b,h). Although the database indicated that *AtSWEET13* and *AtSWEET14* were expressed abundantly in stamens, promoter activity was also detected at other developmental stages with similar expression patterns (Fig. 3c–f,i–l). In seedlings of *pAtSWEET13:GUS* and *pAtSWEET14:GUS* transgenic plants, GUS activity was detected around the vascular tissues in leaves and roots (Fig. 3c,d,i,j). Dot-like GUS staining at the junctions of stems and petioles suggested that *AtSWEET13* and *AtSWEET14* were also expressed in axillary buds (Fig. 3e,k). During seed development, the promoter activity of *AtSWEET13* and *AtSWEET14* was detected in embryonic cotyledons (Fig. 3f,l).

**Figure 4:**
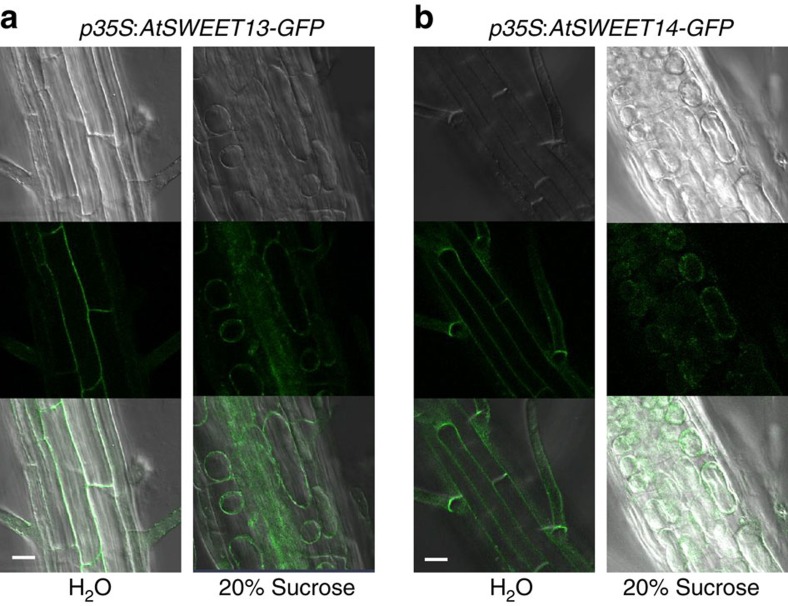
Subcellular localization of AtSWEET13 and AtSWEET14. (**a**) GFP fluorescence in *p35S:AtSWEET13-GFP* transgenic plants. (**b**) GFP fluorescence in *p35S:AtSWEET14-GFP* transgenic plants. For (**a**,**b**), roots of 1-week-old seedlings treated with water (H_2_O) or 20% (w/v) sucrose were used for observations. Upper panels are bright-field images. Middle panels are images of GFP fluorescence. Lower panels are merged images of bright-field and GFP fluorescence. Scale bars, 20 μm.

When AtSWEET13 and AtSWEET14 were expressed as GFP fusion proteins under the control of the 35S promoter, GFP fluorescence was associated with the plasma membrane (Fig. 4).

### AtSWEET13 and AtSWEET14 regulate anther dehiscence

**Figure 5:**
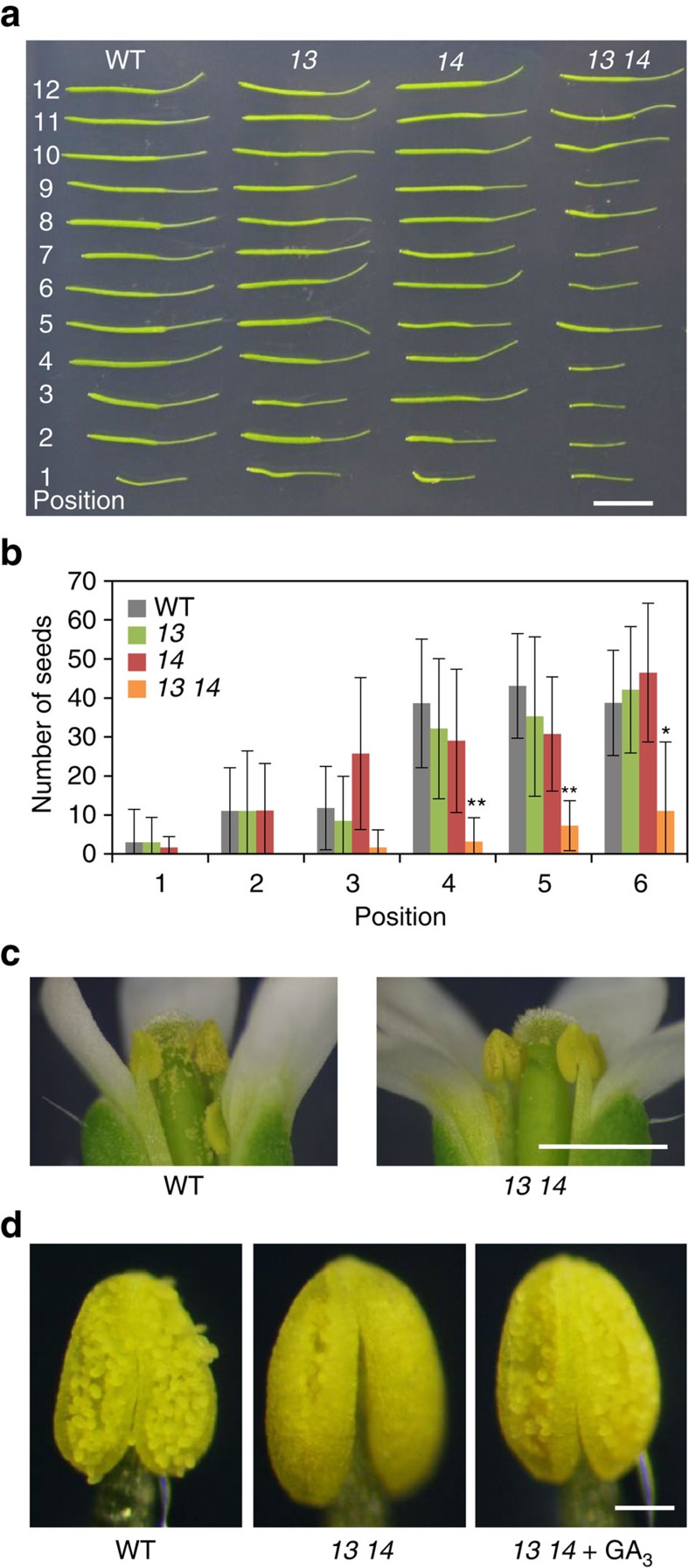
Phenotypes of *sweet13 sweet14* in flowers and siliques. (**a**) Representative siliques from wild type (WT), *sweet13* (*13*), *sweet14* (*14*) and *sweet13 sweet14* (*13 14*). Positions of the siliques from the bottom are indicated on the left side of the photo. Scale bar, 1 cm. (**b**) Numbers of seeds per siliques in wild type (WT), *sweet13* (*13*), *sweet14* (*14*) and *sweet13 sweet14* (*13 14*). Averages of eight siliques are shown with standard deviations. The seed number of siliques at different positions varied significantly; *P<0.05, **P<0.001; Tukey–Kramer test. (**c**) Representative photos of flowers and anthers of wild type (WT) and *sweet13 sweet14*. Scale bar, 1 mm. (**d**) Restoration of the *sweet13 sweet14* phenotype by GA_3_ treatment. Excised flower buds of *sweet13 sweet14* were incubated with water (*13 14*) or 100 μM GA_3_ (*13 14*+GA_3_) and anthers were observed after 1 day. Wild type flower buds incubated with water were also observed for comparison (WT). Scale bar, 0.1 mm.

To delineate the physiological roles of AtSWEET13 and AtSWEET14, we obtained mutants defective in the respective proteins (*sweet13* and *sweet14*). As the expression patterns of *AtSWEET13* and *AtSWEET14* were similar, we generated a double mutant of *sweet13* and *sweet14* (*sweet13 sweet14*). We noted that the double mutant had reduced fertility and produced fewer seeds per silique compared with the wild type, whereas this phenotype was not observed in the *sweet13* and *sweet14* single mutants (Fig. 5a,b). The phenotype was complemented by the introduction of either *AtSWEET13* or *AtSWEET14* genomic DNA (Supplementary Fig. 3), indicating that this phenotype resulted from the double mutation. Expression of *AtSWEET13* and *AtSWEET14* in anthers (Fig. 3) suggested that this phenotype might be related to anther development as was observed for some GA-deficient and signalling mutants^25^. Closer investigation revealed that anther dehiscence was delayed in *sweet13 sweet14* compared with the wild type, whereas filament elongation was similar to the wild type (Fig. 5c). Treatment of flower buds with an excess amount (100 μM) of GA_3_ restored the delayed anther dehiscence of the double mutant (Fig. 5d). Pollen from *sweet13 sweet14* is likely to have developed normally because siliques of the double mutant set at later stages sometimes developed similarly to those of the wild type (Fig. 5a). GA synthesized in stamens has been hypothesized to regulate flower development^25^,^39^,^40^,^41^; however, the phenotype of *sweet13 sweet14* was restricted to anthers. GA content in whole anthers and filaments was similar between wild type and *sweet13 sweet14* plants (Table 1).

### Seed and seedling phenotypes of *sweet13 sweet14*

**Figure 6:**
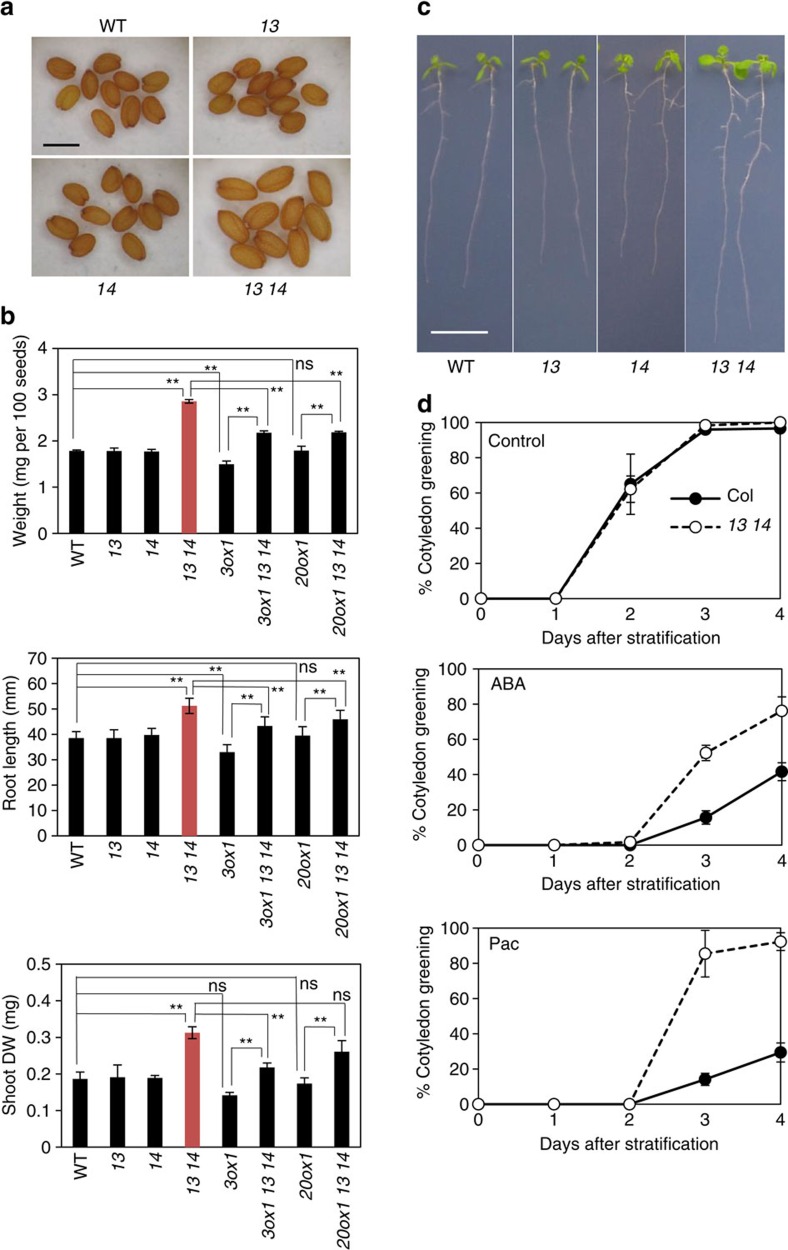
Phenotypes of *sweet13 sweet14* in seeds and seedlings. (**a**) Dry seeds of wild type (WT), *sweet13* (*13*), *sweet14* (*14*), and *sweet13 sweet14* (*13 14*). Scale bar, 0.5 mm. (**b**) Phenotypes of wild type (WT), *sweet13* (*13*), *sweet14* (*14*), *sweet13 sweet14* (*13 14*), *ga3ox1* (*3ox1*), *ga3ox1 sweet13 sweet14* (*3ox1 13 14*), *ga20ox1* (*20ox1*), and *ga20ox1 sweet13 sweet14* (*20ox1 13 14*). Upper panel: weight for 100 dry seeds was measured independently three times for each genotype and the average values are shown with standard deviations. Middle panel: root length of 8-day-old seedlings grown on Murashige and Skoog media without sugars (averages of 40–50 seedlings) is shown with ±s.d.. Lower panel: shoot dry weight of 13–20 seedlings that were grown for 8 days on Murashige and Skoog media without sugars was measured and the weight per shoot was calculated. Values are means±s.d. of three biological replicates. **P<0.001; ns, not significant (*P*>0.05); Tukey–Kramer test. (**c**) Seedlings of (WT), *sweet13* (*13*), *sweet14* (*14*), and *sweet13 sweet14* (*13 14*) grown for 8 days on Murashige and Skoog media without sugars. Scale bar, 1 cm. (**d**) Germination of wild type (WT) and *sweet13 sweet14* (*13 14*) in the presence or absence of 0.5 μM ABA or 10 μM paclobutrazol (Pac). Germination rates of approximately 50 seeds were scored every day and averages of three biological replicates are shown with standard deviations. Cotyledon greening was the indicator of germination.

Promoter–reporter analyses indicated that *AtSWEET13* and *AtSWEET14* were expressed not only in anthers but also in developing seeds and seedlings (Fig. 3). Careful investigation revealed that mature seeds of *sweet13 sweet14* were larger than those of the wild type and were approximately 1.5-fold heavier, whereas this phenotype was not observed in the *sweet13* and *sweet14* single mutants (Fig. 6a,b). Interestingly, in contrast to *sweet13 sweet14*, the *ga3ox1* mutant, which is defective in the GA biosynthesis gene *AtGA3ox1*, produced smaller seeds than the wild type (Fig. 6b). Introduction of the *ga3ox1* mutation reduced the size of the *sweet13 sweet14* double mutant seeds (Fig. 6b). Although the seed size of another GA-deficient mutant *ga20ox1* was not significantly different from the wild type, introduction of the *ga20ox1* mutation clearly reduced the seed size of *sweet13 sweet14* (Fig. 6b). These results indicate that GA is required for the seed size phenotype observed in *sweet13 sweet14*. In *Arabidopsis*, the early 13-hydroxylation pathway to produce GA_1_ as a bioactive GA is dominant during the middle stages of seed development^42^. Although the GA_1_ content in developing seeds at 10 days after flowering (DAF) was comparable between wild type and *sweet13 sweet14*, the levels of deactivated forms of GAs, GA_8_ and GA_29_, were significantly reduced in *sweet13 sweet14* compared with wild type (Table 1). Endogenous levels of other hormones (IAA, ABA, JA, JA-Ile, SA, isopentenyladenine and *trans*-zeatin) in the developing seeds were not extremely different (<1.5-fold) in *sweet13 sweet14* compared with wild type, although there are some statistically significant differences (Supplementary Table 1).

We subsequently found that *sweet13 sweet14* seedlings grew larger than wild-type seedlings and were characterized by longer roots and heavier shoot dry weights (Fig. 6b,c). The phenotype was observed even when 1% sucrose was present in the media (Supplementary Fig. 4). As observed for the seeds, the phenotype of *ga3ox1* seedlings contrasted to that of *sweet13 sweet14* seedlings; *ga3ox1* seedlings had shorter root lengths compared with wild-type seedlings. Introduction of the *ga3ox1* mutation reduced the size of the *sweet13 sweet14* double mutant seedlings. Although the seedling size of *ga20ox1* was comparable to that of wild type, the *ga20ox1* mutation also significantly reduced seedling size in the *sweet13 sweet14* double mutant background. Again, these data suggest that the phenotype observed in *sweet13 sweet14* is dependent on GA. Endogenous levels of GAs and other hormones were quantified in the shoots and roots of the seedlings; however, these hormones accumulated at similar levels in wild type and *sweet13 sweet14* with only a few statistically significant differences (Table 1; Supplementary Table 1).

The phenotypes observed in *sweet13 sweet14* seeds and seedlings were complemented by transformation with either *AtSWEET13* or *AtSWEET14* genomic DNA (Supplementary Fig. 5a,b), indicating that either gene is sufficient for normal seed and seedling development. Vegetative growth in the later developmental stages was comparable between *sweet13 sweet14* and wild type (Supplementary Fig. 6).

As the seed and seedling size phenotype of *sweet13 sweet14* contrasted to that of the GA-deficient mutants, we hypothesized that GA-mediated physiological processes were promoted in the double mutant. GA and ABA are known to have antagonistic effects on seed germination; GA promotes, whereas ABA inhibits seed germination^43^,^44^. Thus, we determined the effects of ABA and the GA biosynthesis inhibitor paclobutrazol on seed germination (Fig. 6d). Both wild type and *sweet13 sweet14* germinated similarly under normal conditions after stratification; however, in the presence of ABA, *sweet13 sweet14* germinated better than wild type. Similarly, *sweet13 sweet14* was more resistant to paclobutrazol in terms of inhibition of seed germination compared with wild type. These results are consistent with GA responses being overactivated in *sweet13 sweet14*.

### Transport of exogenously applied GA

**Figure 7:**
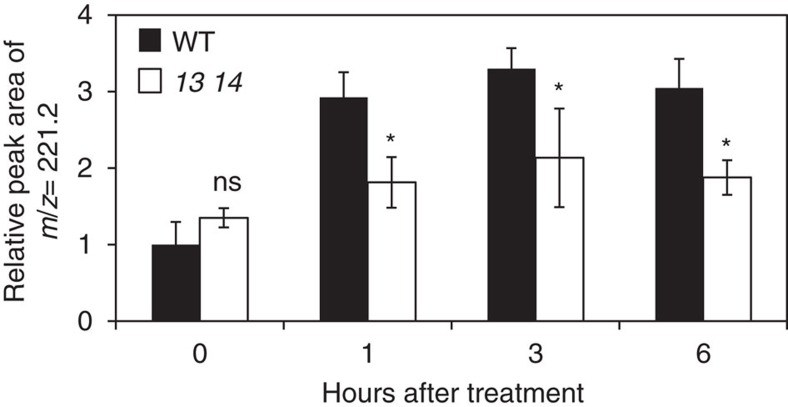
Transport of exogenously applied GA from roots to shoots. The primary root tips of 8-day-old wild-type (WT) and *sweet13 sweet14* (*13 14*) seedlings were spotted with 2μl of 10 μM GA_3_. After incubation for 0, 1, 3 and 6 h, the fragment ion with a *m*/*z* of 221.2 derived from the molecular ion with a *m*/*z* of 345.2 was detected from shoots by LC–MS/MS. Peak areas relative to those of wild type before the treatment (0 h) are shown as means of three biological replicates with standard deviations. *P<0.05; NS, not significant (*P*>0.05); Student's *t*-test.

We then investigated the movement of exogenously applied GA from the roots to the shoots in wild type and *sweet13 sweet14* (Fig. 7). GA_3_ was used as a tracer for this experiment because it is hardly detected in *Arabidopsis* and can be discriminated from endogenous GAs. Droplets of GA_3_-containing water were applied to the main root tip of wild type and *sweet13 sweet14* seedlings, and GA_3_ transported to the shoots was detected by LC–MS/MS. A product ion corresponding to GA_3_ was detected at background levels both in wild type and *sweet13 sweet14* shoots even before the treatment. In wild type, the relative peak area of the product ion corresponding to GA_3_ increased within 1 h after application of GA_3_ and the level was maintained for 6 h. In *sweet13 sweet14*, however, the fragment ion corresponding to GA_3_ was detected at significantly lower levels compared with wild type after GA_3_ treatment. This result indicates that GA_3_ exogenously applied to roots was transported to shoots less efficiently in *sweet13 sweet14* compared with wild type.

## Discussion

Using a Y2H system with the GA receptor GID1a and the DELLA protein GAI, we identified *Arabidopsis* SWEET proteins as novel candidate GA transporters. Among the six closely related AtSWEET proteins tested, AtSWEET10, AtSWEET12, AtSWEET13 and AtSWEET14 were found to enhance GA-dependent interactions between GID1a and GAI in yeast (Fig. 1), suggesting that these proteins mediate GA uptake into yeast cells. Among these, AtSWEET13 and AtSWEET14 are close homologues and their spatial gene expression patterns were predicted to be similar. Thus, we hypothesized that AtSWEET13 and AtSWEET14 play similar physiological roles in plants and examined their functions in more detail.

Although the sucrose transport activities of AtSWEET13 and AtSWEET14 were reported previously^27^, we confirmed that AtSWEET13 and AtSWEET14 could mediate cellular GA uptake when expressed in yeast or *Xenopus* oocytes using mass spectrometry (Fig. 2). The sugar transport activities of SWEET proteins are independent of pH^26^,^27^. In our study, the GA transport activities of AtSWEET13 and AtSWEET14 were dependent on pH (Supplementary Fig. 2a), indicating that these proteins are proton symporters or that protonation at the carboxyl group at the C6 position of GA is required for substrate recognition. In yeast, GA transport activities of AtSWEET13 and AtSWEET14 required glucose (Supplementary Fig. 2a); however, the effect was not observed when examined in *Xenopus* oocytes (Supplementary Fig. 2c). In addition, the GA transport activity of AtNPF2.5 expressed in yeast was also promoted by glucose (Supplementary Fig. 2b). These results indicate that the GA transport activities of AtSWEET13 and AtSWEET14 are not directly regulated by glucose. The reason for this observation is unknown, but possibly glucose inhibits the degradation of GA in yeast or non-specific exclusion, for example mediated by multi-drug resistance transporters.

SWEET proteins are thought to function as low affinity sugar transporters because the *K*m values of previously characterized SWEET proteins for sugars were in the mM range^26^,^27^,^30^,^31^. Given that endogenous GA levels are much lower than that of sugars, the affinities of AtSWEET13 and AtSWEET14 for GA are low even though the *K*m values of AtSWEET13 and AtSWEET14 for GA are lower than those for sugars (Supplementary Fig. 2f,g). Nevertheless, AtSWEET13 and AtSWEET14 induced BD-GID1a/AD-GAI interactions in yeast even at GA concentrations of 1 nM (Fig. 1). Therefore, it is still possible that AtSWEET13 and AtSWEET14 could mediate GA transport *in vivo*. Although AtSWEET13 and AtSWEET14 transported several GA species irrespective of their biological activities (Fig. 2d), they did not transport other plant hormones such as IAA, ABA, JA and JA-Ile (Fig. 2e). This finding indicates that AtSWEET13 and AtSWEET14 are able to specifically recognize a common structure present in GAs.

Promoter–reporter analyses showed that *AtSWEET13* and *AtSWEET14* are expressed in stamens (Fig. 3), and the fertility of the *sweet13 sweet14* double mutant was reduced and anther dehiscence was delayed (Fig. 5). This phenotype was reversed by exogenous application of an excess amount of GA (Fig. 5). The stamen is thought to be an active site for GA biosynthesis^25^,^39^,^40^,^41^,^45^,^46^, and severe mutants impaired in GA biosynthesis or signalling have defective stamen development and reduced fertility^24^,^25^. Despite the fact that GA regulates multiple steps during stamen development including pollen maturation, filament elongation and anther dehiscence, the *sweet13 sweet14* phenotype was restricted to anther dehiscence. This is in contrast to mutants lacking AtNPF2.10/GTR1 (ref. 18) suggesting that these transporters may be required for different processes in stamen development. Although the phenotype of *sweet13 sweet14* resembled GA-deficient mutants in part, the bulk GA content in anthers was comparable in wild type and *sweet13 sweet14* (Table 1).

Based on the transport activity seen in yeast and oocyte-based assays and the plasma membrane localization of AtSWEET13 and AtSWEET14 we propose that these proteins may mediate GA uptake into cells. This may in turn influence the local distribution of GA within anthers. Therefore, the loss of AtSWEET13 and AtSWEET14 function possibly results in reduced cellular GA levels without affecting the bulk GA content.

GA synthesized in stamens regulates flower development^25^,^39^,^40^,^41^; however, we do not believe that AtSWEET13 and AtSWEET14 are involved in this process because flower tissues, other than the anthers, appeared normal in *sweet13 sweet14* plants (Fig. 5c). AtSWEET13 and AtSWEET14 transported not only bioactive GAs but also their precursors and metabolites in yeast (Fig. 2d); however, the membrane permeability of GAs decreased, probably due to increased hydrophilicity after successive oxidation by GA13ox, GA2ox and GA3ox. This result suggests that GA_1,_ rather than GA_4_ and their precursors, requires specific transporters for translocation across biological membranes. GA_4_ is the major bioactive GA in flower tissues, including anthers in rice, petunia and pumpkin^39^,^40^,^45^, and was observed in whole flowers from wild-type *Arabidopsis* (Table 1). Interestingly, GA_1_ accumulated predominantly in anthers, compared with GA_4_ (Table 1). Based on this data we suggest that GA_1_ might be the predominant substrate of AtSWEET13 and AtSWEET14 in anthers. Nevertheless, we cannot exclude the possibility that defective anther development in *sweet13 sweet14* is not due to altered GA transport. For example, it is possible that alterations in sugar transport effect anther development.

We found that the seeds produced by *sweet13 sweet14* were larger than those produced by the wild type (Fig. 6a,b). The roles of GA on seed size have not been discussed in previous studies, but our study demonstrated that *ga3ox1* produced smaller seeds than did the wild type (Fig. 6b). In contrast, the seed size phenotype was not obvious in *ga20ox1* mutants (Fig. 6b), possibly due to different degrees of redundancies between *ga3ox1* and *ga20ox1*. Nevertheless, mutations in either *GA3ox1* or *GA20ox1* reduced seed size in the *sweet13 sweet14* mutant background, indicating that GA is required for the phenotype observed in *sweet13 sweet14*. Therefore, the larger size of *sweet13 sweet14* seeds might be attributed to enhanced GA responses. Again, we cannot exclude the possibility that the larger seed size of *sweet13 sweet14* is unrelated to differences in GA transport. For example, it could potentially have been caused by enhanced carbohydrate translocation from source tissues. However, sugars inhibit seed germination and/or early seedling growth^47^, a condition that is not consistent with the observation that the double mutant germinated well in the presence of ABA or paclobutrazol compared with the wild type (Fig. 6d).

We have previously shown that GA_1_ is the major bioactive GA present in the middle stage of seed development^42^. As mentioned above, GA_1_ may require specific transporters to be translocated across biological membranes. Although the GA_1_ content in developing seeds was comparable between wild type and *sweet13 sweet14,* the levels of GA_8_ and GA_29_ (2-oxidation products of GA_1_ and its precursor GA_20_, respectively) were significantly reduced in *sweet13 sweet14* compared with wild type (Table 1). It is not clear why these levels were altered but we speculate that deactivation pathways might be activated due to enhanced GA response.

Seedling size was larger in *sweet13 sweet14* mutants. As GA is a positive regulator of vegetative growth, and mutants defective in GA biosynthesis and signalling often exhibit dwarfism^24^, this is consistent with an over-response to GA during early seedling growth in the mutant, as discussed above in relation to seed development and germination. Promoter–reporter analyses suggested that vascular tissues are potential sites of GA biosynthesis in seedlings^24^ and promoter activities of *AtSWEET13* and *AtSWEET14* were also detected around the vascular tissues in shoots and roots (Fig. 3). Less GA_3_ was transported to *sweet13 sweet14* shoots compared with wild-type shoots when exogenously applied to root tips (Fig. 7). We hypothesize that AtSWEET13 and AtSWEET14 may be associated with GA transported in or out of the vascular tissues and that exogenously applied GA_3_ is first taken into the cells of vascular tissues where AtSWEET13 and AtSWEET14 are expressed, and then transported to the xylem to be transported to shoots. GA is thought to accumulate in the root endodermis^48^; however, it is unclear whether AtSWEET13 and AtSWEET14 are involved in these processes. GA_4_ is the major bioactive form in *Arabidopsis* seedlings (Table 1). Whether AtSWEET13 and AtSWEET14 transport GA_4_ or its precursors, despite their relatively high membrane permeabilities, is unknown. Despite not accumulating to detectable levels it is also possible that AtSWEET13 and AtSWEET14 regulate the transport of GA_1_ in seedlings. As above, we cannot exclude that the seedling phenotypes and altered transport of exogenous GA are indirect. They may for example be related to alterations in sugar transport. These possibilities must be clarified in future studies.

In conclusion, we demonstrated that SWEET proteins are multifunctional transporters. Among the 17 homologues present in the *Arabidopsis* SWEET family, some members might transport compounds other than GA and sugars. Interestingly, multifunctionality has recently been reported for NPF^11^,^17^. Further elucidation of novel transporter functions will explain how plants are able to transport a variety of compounds with only a limited number of transporters.

## Methods

### Yeast screening

The BD-GID1a and AD-GAI Y2H system^17^ was used to screen for a cDNA library constructed from RNA extracted from 2-week-old *Arabidopsis* seedlings^16^. Colonies that grew on selection medium (synthetic dextrose (SD); -Leu, -Trp, -Ura, -His) in the presence of 0.1 μM GA_3_ were selected.

### Cloning *AtSWEET* cDNA

Coding sequences of six *AtSWEET* cDNAs were amplified by PCR using the following primer combinations: 5′-CACCATGTTCCTCAAGGTTCATGAAATTGC-3′ (forward) and 5′-TCACTTCATTGGCCTCACCG-3′ (reverse) for *AtSWEET9* (AT2G39060), 5′-CACCATGGCAATTTCACAAGCCGTC-3′ (forward) and 5′-TTAATTCTTAGAAATGAGAAATACTTCTTTTTCATCC-3′ (reverse) for *AtSWEET10* (AT5G50790), 5′-CACCATGAGTCTCTTCAACACTGAAAACAC-3′ (forward) and 5′-TCATGTAGCTGCTGCGGAAG-3′ (reverse) for *AtSWEET11* (AT3G48740), 5′-CACCATGGCTCTCTTCGACACTCATAA-3′ (forward) and 5′-TCAAGTAGTTGCAGCACTGTTTC-3′ (reverse) for *AtSWEET12* (AT5G23660), 5′-CACCATGGCTCTAACTAACAATTTATGGGC-3′(forward) and 5′-TTAAACTTGACTTTGTTTCTGGACATCC-3′ (reverse) for *AtSWEET13* (AT5G50800), and 5′-CACCATGGTTCTCACTCACAACGTATTGGC-3′ (forward) and 5′-TTAGTTTGGCATTTTCTTGTCCATC-3′ (reverse) for *AtSWEET14* (AT4G25010). Underlined sequences denote overhangs that were used to clone the sequence into pENTR/D-TOPO (Invitrogen).

### Transport assays in yeast

For the Y2H-based indirect assays, *AtSWEET* cDNAs were cloned into a yeast expression vector pYES-DEST52 (Invitrogen), in which the *GAL1* promoter had been replaced with the *ADH1* promoter by LR reactions^16^. BD-GID1a/AD-GAI interactions were detected based on the expression of *HIS3* or *LacZ* markers.

For direct assays in yeast, *AtSWEET13, AtSWEET14* or *AtNPF2.5* cDNA cloned in pYES-DEST52 was transformed into the yeast strain INV*Sc*1 (Invitrogen). As a negative control, the empty pYES-DEST52 vector was transformed. *AtNPF2.5* cDNA was amplified using primers 5′-CACCATGGCTGATTCAAAATCTGG-3′ (forward) and 5′-CTAGGTTTTAACATCTTTAG-3′ (reverse) and cloned into pENTR/D-TOPO^17^ and then into pYES-DEST52. Underlined sequence denotes overhangs that were used to clone the sequence into pENTR/D-TOPO. Precultured yeast cells were cultured overnight in medium (SD; -Ura), and cells were collected when the OD_600_ reached 0.7 to 1.0. Media containing 2% (w/v) galactose and 1% (w/v) raffinose instead of 2% (w/v) glucose were used for the culture. Cells were washed with 50 mM potassium phosphate buffer (KPB; pH 5.8) and the OD_600_ was adjusted to 10 with 50 mM KPB (pH 5.8). The cell suspension (0.4 ml) was added to 0.6 ml KPB (pH 5.8) containing 1 μl hormone stock solution (at 1,000 × concentrations in DMSO) and incubated at 25 °C. After incubation, cells were collected by centrifugation at 15,000*g* for 1 min and then washed three times with 1 ml of 50 mM KPB (pH 5.8). We defined the end of the reaction as the first wash with KPB. The cells were stored at −80 °C until extraction. Quantification of hormones by LC–MS/MS was performed using deuterium labelled compounds as internal standards (see below).

### Transport assays in *Xenopus* oocytes

*AtSWEET13* and *AtSWEET14* cDNAs were amplified with following primers: 5′-TTTGAATTCATGGCTCTAACTAACAATTT-3′ (forward)/5′-TTTGGATCCTTAAACTTGACTTTGTTTCT-3′ (reverse) for *AtSWEET13*, and 5′-TTTGAATTCATGGTTCTCACTCACAAC-3′ forward)/5′- TTTGGATCCTTAGTTTGGCATTTTCTT-3′ (reverse) for *AtSWEET14*. The amplification products were subcloned into the *Eco*RI and *Bam*HI sites of the plasmid to synthesize cRNAs^49^. Capped cRNA of each gene was prepared using the mMESSAGE mMACHINE kit (Life Technologies). Each oocyte was injected with 500 ng cRNA. After incubation for 24 h, the buffer was replaced with 100 μl Kulori-based solution (90 mM sodium gluconate, 1 mM potassium gluconate, 1 mM calcium gluconate, 1 mM magnesium gluconate, 1 mM LaCl_3_ and 10 mM MES, pH 5.0) containing substrates. Substrate concentrations and incubation time are indicated in each figure legend. Oocytes were washed twice with 200 mM sorbitol and homogenized in 40 μl extraction buffer (28% methanol, 0.05% acetic acid). After incubated at 4 °C for 24 h, samples were centrifuged at 20,000*g* at room temperature for 20 min and supernatants were collected. Samples (10 μl) were subjected to ultra-performance liquid chromatography (UPLC) coupled to time-of-flight mass spectrometry (TOFMS)^18^. An Agilent 1290 Infinity (Agilent Technologies) equipped with a ZORBAX Eclipse Plus C18 column (1.8 mm, 2.1 × 50 mm; Agilent Technologies) and a micrOTOF II (Bruker Daltonics) were used for the analysis. The mobile phases used for UPLC were as follows: A, 20% (v/v) aqueous methanol with 0.05% (v/v) acetic acid, and B, methanol with 0.05% (v/v) acetic acid. The gradient program was as follows: 0 to 3.5 min, isocratic 90% A; 3.5 to 6 min, linear gradient 90 to 0% A; 6.1 min to 9 min, isocratic 90% A, with a flow rate of 0.15 ml min^−1^. The TOFMS analysis was performed in the negative mode with scan range of 100–700 *m*/*z*. The capillary voltage was set at 4,200 V, the nebulizer gas pressure was set at 1.6 bar, the desolvation gas flow was set at 8.0 l min^−1^, and the temperature was set at 180 °C. GA levels were quantified based on extracted ion chromatograms and the corresponding peak position of the standard compound.

### Chemicals

ABA, IAA and D_2_-IAA were purchased from Sigma-Aldrich. GA_3_ was purchased from Wako. JA, JA-Ile and ^13^C_6_-JA-Ile (ref. 50) were gifted from Dr. Yusuke Jikumaru (RIKEN). D_2_-JA was purchased from Tokyo Kasei. D_6_-ABA was purchased from Icon Isotope. D_2_-GAs, D_5_-tZ, D_6_-iP and D_3_-dihydrozeatin were purchased from OlChemIm. D_6_-SA was purchased from Isotec.

### Plant materials

Homozygous *sweet13* (SALK_087791) and *sweet14* (SALK_010224) mutants were selected by PCR using primers designed according to the SIGnAL (Salk Institute Genomic Analysis Laboratory) website (http://signal.salk.edu/tdnaprimers.2.html). A homozygous *ga20ox1* mutant (*ga20ox1-3*; SALK_016701C)^51^ was obtained from the *Arabidopsis* Biological Resource Center (ABRC). The *ga3ox1* mutant (*ga3ox1-3*)^52^ was a gift from Dr. Shinjiro Yamaguchi (Tohoku University).

To generate *pAtSWEET13*:*GUS* and *pAtSWEET14*:*GUS* lines, approximately 2 kb upstream regions from the start codon were amplified by PCR and cloned into pENTR/D-TOPO (Invitrogen). After the DNA sequences were confirmed, the promoter regions were cloned into pGWB3 (ref. 53) by LR reactions. Primer combinations used to amplify *AtSWEET13* and *AtSWEET14* promoter regions were as follows; 5′-CACCACCCTTTCTAAAACACAAGTTTATGG-3′ (forward)/5′-TTCTTGATGTTTCTTGATTAGTTTTCTCTC-3′ (reverse), and 5′-CACCAGTTTAAAAACAAGGGAGACAGC-3′ (forward)/5′-TTTGGTTATTTTGTGAATGAGCGAG-3′ (reverse), respectively. Underlined sequences in the primers identify overhangs that were used for cloning into pENTR/D-TOPO. Each construct was introduced into *Agrobacterium* strain GV3101 and transformed into the *Arabidopsis* Col-0 accession by the floral-dip method. The staining buffer used to detect GUS expression was composed of 50 mM sodium phosphate buffer (pH 7.2), 10 mM EDTA, 0.05% (v/v) Triton X-100, 0.5 mM potassium ferrocyanide, 0.5 mM potassium ferricyanide and 1 mM X-Gluc.

To analyse the subcellular location of AtSWEET13 and AtSWEET14, transgenic plants expressing C-terminus GFP fusion proteins under the control of the cauliflower mosaic virus (CaMV) 35S promoter were produced. Coding sequences for *AtSWEET13* and *AtSWEET14* without a stop codon were amplified by PCR using the primer combinations:5′-CACCATGGCTCTAACTAACAATTTATGGGC-3′ (forward)/5′-AACTTGACTTTGTTTCTGGAC-3′ (reverse) and 5′-CACCATGGTTCTCACTCACAACGTATTGGC-3′ (forward)/5′-GTTTGGCATTTTCTTGTCCATC-3′ (reverse), respectively, and were cloned into pENTR/D-TOPO. Underlined sequences in the primers identify overhangs that were used for cloning into pENTR/D-TOPO. After the DNA sequences were confirmed, the coding sequences were cloned into pGWB5 (ref. 53) by LR reactions. Each construct was introduced into *Agrobacterium* strain GV3101 and transformed into *Arabidopsis* Col-0 accession by the floral-dip method. GFP fluorescence was observed by confocal laser scanning microscopy (LSM700, Carl Zeiss).

For complementation of *sweet13 sweet14*, the *AtSWEET13* genomic sequence containing its promoter region was amplified with the following primers: 5′-ACCCTTTCTAAAACACAAGTTTATGG-3′ (forward) and 5′-TTAAACTTGACTTTGTTTCTGGACATCC-3′ (reverse) of which the 5′ ends were phosphorylated. The amplified fragments were cloned into pBIB-HYG that had been digested with *Sma*I and dephosphorylated, and the DNA sequences of the inserted fragments were confirmed. The *AtSWEET14* genomic sequence including the promoter region was amplified with the primers 5′-GTCGACAGTTTAAAAACAAGGGAGACAGC-3′ (forward) and 5′-CCCGGGTTAGTTTGGCATTTTCTTGTCCATCTG-3′ (reverse) and cloned into pCR-Blunt II-TOPO. Underlined sequences in the primers identify restriction sites. After the DNA sequences were confirmed, the inserted fragment was excised by digestion with *Sal*I and *Sma*I, and then cloned into *Sal*I-*Sma*I-digested pBIB-HYG. Each construct was introduced into *Agrobacterium* strain GV3101 and transformed into *Arabidopsis* Col-0 accession by the floral-dip method.

### Plant growth conditions

*Arabidopsis* seeds were surface-sterilized in a solution containing 5% (v/v) NaClO and 0.05% (v/v) Tween 20, rinsed with water, and sown on Murashige and Skoog media (one-half strength Murashige and Skoog salts, MES (0.5 g l^−1^), pH 6.5) containing 0.8% (w/v) agar. After stratification at 4 °C in the dark for 3 days, plates were transferred to continuous light conditions at 22 °C. One-week-old seedlings were transplanted to soil containing vermiculite and Metro-mix 350 (Sun Gro Horticulture) at a 3:1 ratio, supplied with nutrients (1/1,000 dilution of Hyponex; Hyponex Japan) and grown in growth chambers at 22 °C under continuous white light (approximately 10 W m^−2^).

To observe seedlings (Fig. 6), surface-sterilized seeds were germinated on 1.5% (w/v) square agar plates containing Murashige and Skoog medium. After stratification at 4 °C in the dark for 3 days, plates were incubated vertically under continuous light conditions at 22 °C.

For germination assays, surface-sterilized seeds were sown on Murashige and Skoog media containing 0.8% (w/v) agar plates. ABA and paclobutrazol stock solutions (1,000 × concentration) prepared in DMSO were added to the cooled media after autoclaving. Plates containing 0.1% (v/v) DMSO were used for control experiments. After stratification at 4 °C in the dark for 3 days, plates were transferred to continuous light conditions at 22 °C. Germination was defined as cotyledon greening.

To restore the delayed anther dehiscence of *sweet13 sweet14* by GA treatment, main inflorescence stems containing flower buds were excised (approximately 3–4 cm) and the bottom ends were placed in water containing 100 μM GA_3_. After incubation for 1day, anthers were observed with a stereomicroscope (Leica MZ FLIII).

To observe the movement of exogenously applied GA_3_ from roots to shoots, surface-sterilized seeds were germinated on 1.5% (w/v) square agar plates containing Murashige and Skoog medium. After stratification at 4 °C in the dark for 3 days, plates were incubated vertically under continuous light conditions at 22 °C for 8 days. Two μl of a 10 μM GA_3_ solution containing 0.01% (w/v) agar were spotted onto the main root tip. After incubation for 0, 1, 3 and 6 h, fragment ions corresponding to GA_3_ were detected by LC–MS/MS as detailed below.

### Hormone analysis by LC–MS/MS

To determine levels of GA in anthers and filaments, flowers at stage 13 (ref. 54) were dissected using a stereomicroscope. The GA levels in whole flowers at approximately stage 13 were also analysed. To measure the level of hormones in seedlings, 8-day-old plants grown on vertical plates were separated into shoots and roots. To collect developing seeds, siliques at 10 DAF were dissected using a stereomicroscope. All samples were frozen in liquid nitrogen and weighed after lyophilisation.

The samples were ground and homogenized in extract solution (Supplementary Table 2) with defined amounts of deuterium-labelled internal standards. The mixtures were incubated for 12 h at 4 °C and then centrifuged at 3,000*g* for 20 min at 4 °C. The supernatants were dried in a vacuum and dissolved in 1 ml of water containing 1% (v/v) acetic acid. After several steps of purification on solid phase columns, extracts were dried in a vacuum and dissolved in 20 μl of water containing 1% (v/v) acetic acid. The purification steps are summarized in Supplementary Tables 2 and 3. The LC–MS/MS system consisting of a quadrupole/time-of-flight tandem mass spectrometer (Triple TOF 5600, AB SCIEX), and a Nexera HPLC system (SHIMADZU) were used in these analyses. LC separations were performed at a flow rate of 400 μl min^−1^ using the conditions presented in Supplementary Table 4. MS/MS conditions are presented in Supplementary Table 5. We used a software tool (MultiQuant 2.0, AB SCIEX) to calculate plant hormone concentrations from the LC–MS–MS data.

### Data availability

The authors declare that all other data supporting the findings of this study are available within the article and its Supplementary Information files or are available from the corresponding author upon request.

## Additional information

**How to cite this article:** Kanno, Y. *et al*. AtSWEET13 and AtSWEET14 regulate gibberellin-mediated physiological processes. *Nat. Commun.* **7,** 13245 doi: 10.1038/ncomms13245 (2016).

## Supplementary Material

Supplementary Information Supplementary Figures 1-6, Supplementary Tables 1-5 and Supplementary References

## Figures and Tables

**Table 1:**
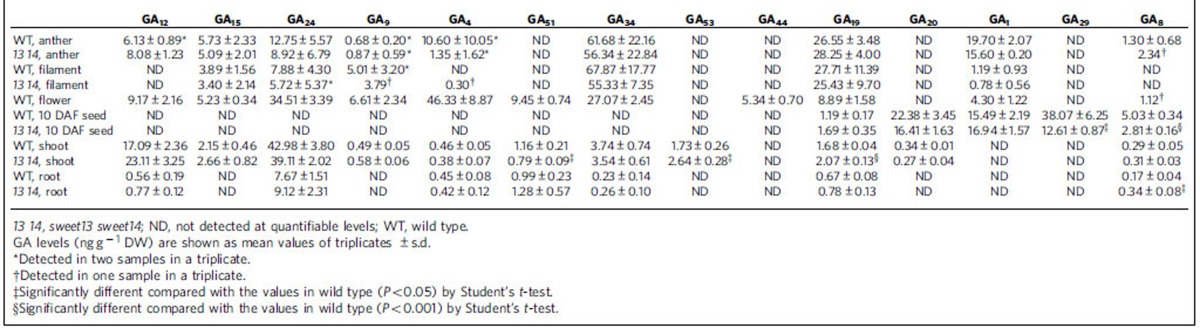
GA levels in wild type and *sweet13 sweet14.*
