# Supplementary material for: AtSWEET13 and AtSWEET14 regulate gibberellin-mediated physiological processes
Source: Nat Commun. 2016 Oct 26;7:13245. doi: 10.1038/ncomms13245 (PMC5095183; doi:10.1038/ncomms13245)
Supplement: Supplementary Information — Supplementary Figures 1-6, Supplementary Tables 1-5 and Supplementary References [file ncomms13245-s1.pdf]

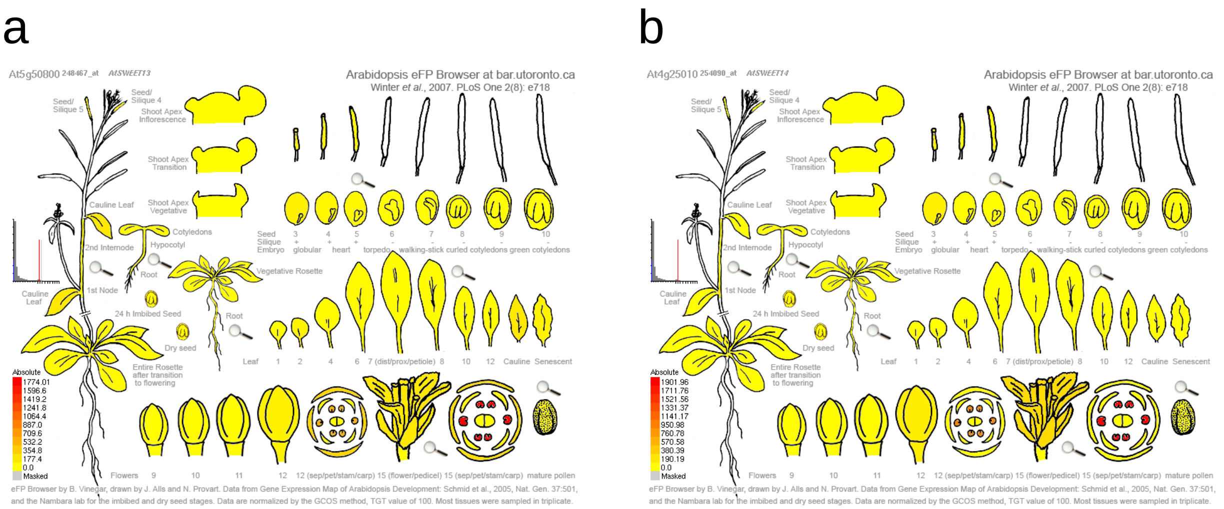

Supplementary Figure 1. Expression of *AtSWEET13* and *AtSWEET14*. (**a**) Expression of *AtSWEET13* and (**b**) *AtSWEET14* obtained from Arabidopsis eFP Browser (http://bar.utoronto.ca/efp/cgi-bin/efpWeb.cgi)[1-3].

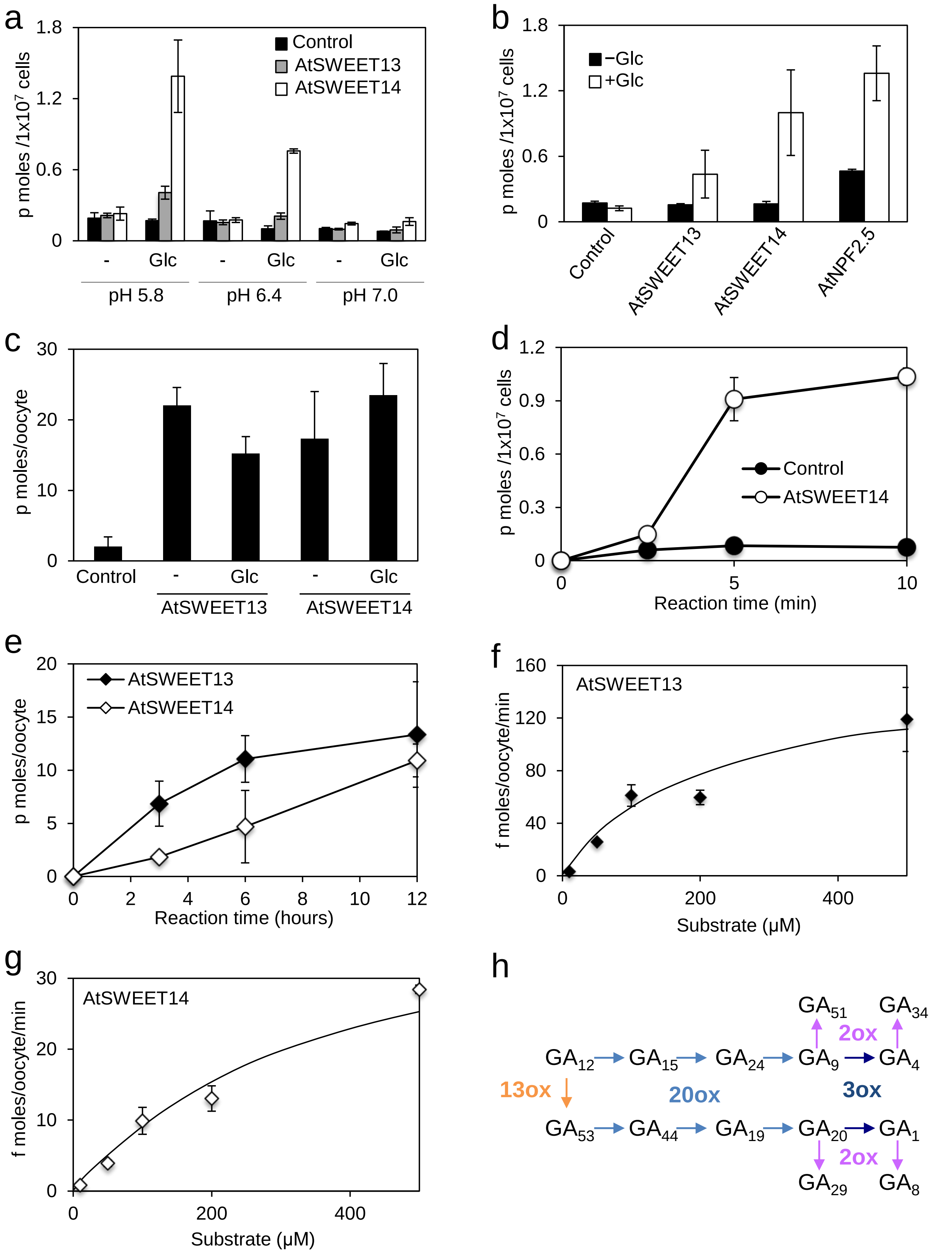

Supplementary Figure 2

Supplementary Figure 2. GA transport activities of AtSWEET13 and AtSWEET14. (**a**) Glucose and pH dependent GA transport activities of AtSWEET13 and AtSWEET14 in yeast. Yeast cells expressing AtSWEET13 or AtSWEET14 were incubated with 10 μM GA_3 in the absence (−) or presence of 100 mM glucose (Glc) at pH 5.8, 6.4 and 7.0. Yeast cells transformed with an empty vector were used as a control. GA_3 taken into cells after 5 min of incubation was analysed by LC-MS/MS. Values are means ± SD of three biological replicates. The data at pH5.8 with glucose is the same as that presented in Fig. 1a. (**b**) Glucose-dependent GA transport activities of AtSWEET13, AtSWEET14 and AtNPF2.5 in yeast. Yeast cells expressing AtSWEET13, AtSWEET14 or AtNPF2.5 were incubated with 10 μM GA_3 in the absence (−Glc) or presence (+Glc) of 100 mM glucose at pH 5.8. Yeast cells transformed with an empty vector were used as a control. GA_3 taken into cells after 10 min of incubation was analysed by LC-MS/MS. Values are means ± SD of three biological replicates. (c) Glucose-dependent GA transport activities of AtSWEET13 and AtSWEET14 in *Xenopus* oocytes. Oocytes injected with *AtSWEET13* or *AtSWEET14* cRNA were incubated for 24 h in Kulori medium-based buffer (pH 5.0) with (Glc) or without (−) 100 mM glucose containing 100 μM GA_3. As a control, water was injected into the oocytes. Values are means ± SD of three or four biological replicates with two oocytes. (**d**) Time-dependent GA uptake into yeast mediated by AtSWEET14. Yeast cells expressing AtSWEET14 were incubated with 10 μM GA_3 in the presence of 100 mM glucose at pH 5.8 for 0, 2.5, 5 and 10 min. Yeast cells transformed with an empty vector were used as a control. Values are means ± SD of three biological replicates. (**e**) Time-dependent GA uptake into *Xenopus* oocytes mediated by AtSWEET13 and AtSWEET14. Oocytes injected with *AtSWEET13* or *AtSWEET14* cRNA were incubated in Kulori medium-based buffer (pH 5.0) containing 100 μM GA_3 for 0, 3, 6, 12 and 24 h. Values are means ± SD of three replicates with two oocytes. (**f**) Concentration-dependent GA uptake into *Xenopus* oocytes mediated by AtSWEET13. Oocytes injected with *AtSWEET13* cRNA were incubated for 3 h in Kulori medium-based buffer (pH 5.0) containing 10, 50, 100, 200 and 500 μM GA_3. Values are means ± SD of three replicates with two oocytes. (**g**) Concentration-dependent GA uptake into *Xenopus* oocytes mediated by AtSWEET14. Oocytes injected with *AtSWEET14* cRNA were incubated for 6 h in Kulori medium-based buffer (pH 5.0) containing 10, 50, 100, 200 and 500 μM GA_3. Values are means ± SD of three replicates with two oocytes. (**h**) Simplified GA metabolic pathway. 13ox, GA 13-oxidase (GA13ox); 20ox, GA 20-oxidase (GA20ox); 3ox, GA 3-oxidase (GA3ox); 2ox, GA 2-oxidase (GA2ox).

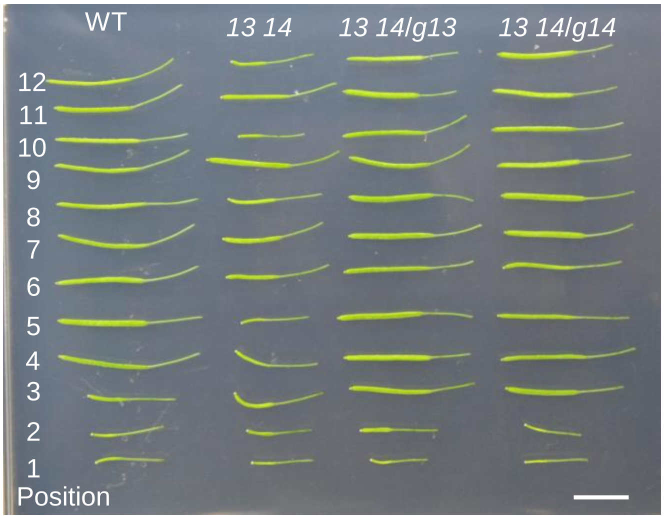

Supplementary Figure 3. Complementation of the *sweet13 sweet14* phenotype in siliques by genomic *AtSWEET13* or *AtSWEET14* DNA. Representative siliques from wild type (WT), *sweet13 sweet14* (*13 14*), *sweet13 sweet14* transformed with *AtSWEET13* genomic DNA (*13 14/g13*), and *sweet13 sweet14* transformed with *AtSWEET14* genomic DNA (*13 14/g14*). Positions of the siliques from the bottom are indicated on the left side of the photo. Scale bar:1 cm.

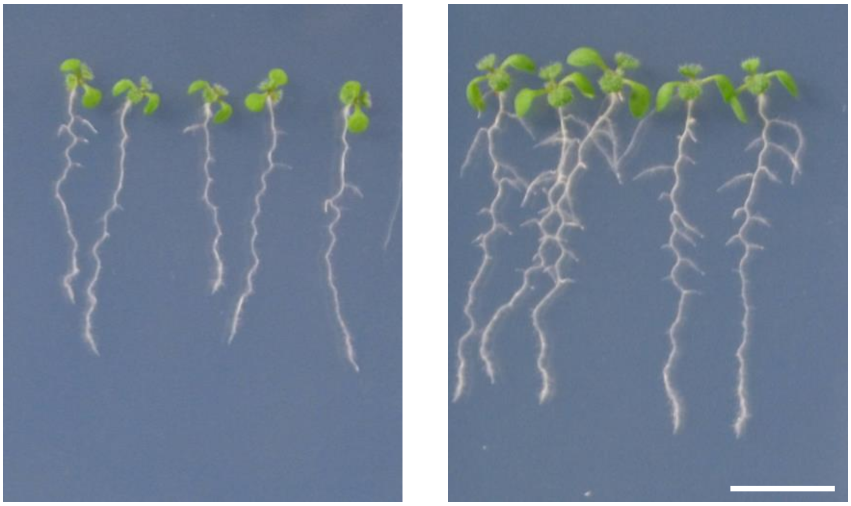

WT *sweet 13 sweet14*

Supplementary Figure 4. Seedling growth on sugar-containing media. Eight-day-old seedlings of wild type (WT) and *sweet13 sweet14* grown on Murashige and Skoog media containing 1% (w/v) sucrose. Scale bar:1 cm.

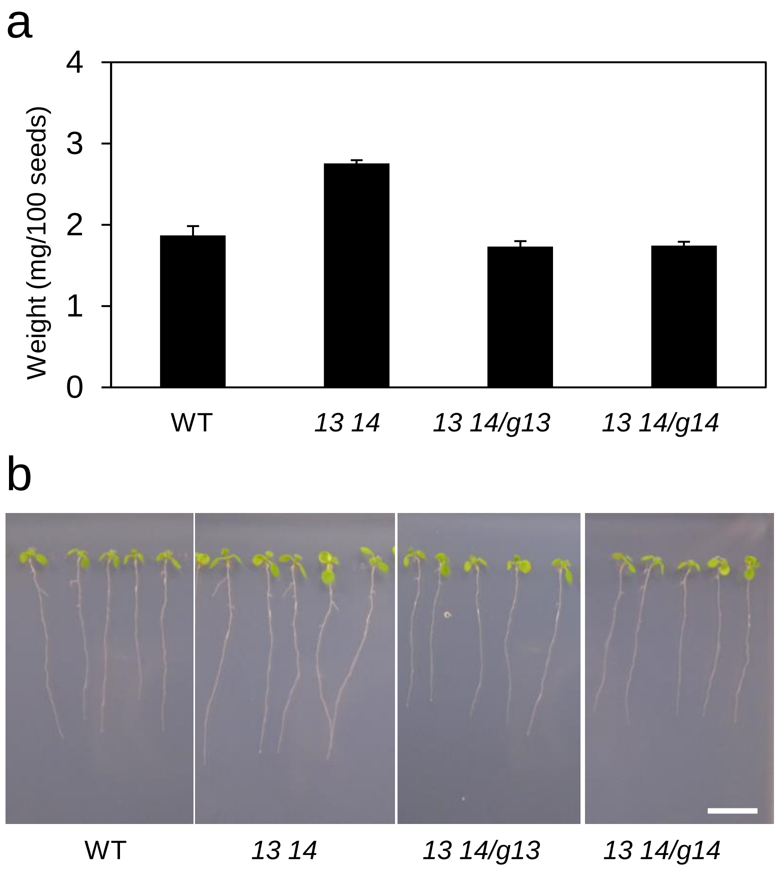

Supplementary Figure 5. Complementation of the *sweet13 sweet14* phenotype in seeds and seedlings by genomic *AtSWEET13* or *AtSWEET14* DNA. (**a**) Seed weight of wild type (WT), *sweet13 sweet14* (*13 14*), *sweet13 sweet14* transformed with *AtSWEET13* genomic DNA (*13 14/g13*), and *sweet13 sweet14* transformed with *AtSWEET14* genomic DNA (*13 14/g14*). Weight of 100 seeds was measured independently three times for each genotype and the averages are shown with standard deviations. (**b**) Seedlings of wild type (WT), *sweet13 sweet14* (*13 14*), *sweet13 sweet14* transformed with *AtSWEET13* genomic DNA (*13 14/g13*), and *sweet13 sweet14* transformed with *AtSWEET14* genomic DNA (*13 14/g14*). Five representative seedlings for each genotype are shown. Scale bar: 1 cm.

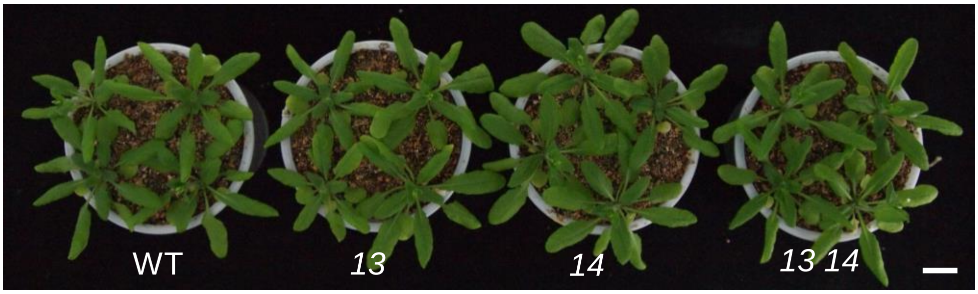

Supplementary Figure 6. Vegetative growth of *sweet13 sweet14*. Representative plants (approximately 1 month old) of wild type (WT), *sweet13* (*13*), *sweet14* (*14*), and *sweet13 sweet14* (*13 14*) comparing vegetative growth. Scale bar: 1 cm.

Supplementary Table 1. Hormone levels in wild type and *sweet13 14.*

	IAA	ABA	JA	JA-Ile	SA	iP	tZ	DHZ
WT, 10 DAF seed	999.89 ±200.47	496,88 ±57.10	407.68 ±45.95	32.16 ±3.40	282.81 ±25.29	0.50 ±0.06	3.42 ±0.26	nd
13 14, 10 DAF seed	785.08 ±24.59	377.09 ±22.20*	533.51 ±52.66*	45.09 ±2.40**	361.57 ±38.25*	0.67 ±0.03*	3.60 ±0.66	nd
WT, shoot	112.93 ±43.63	16.85 ±1.79	7.81 ±4.98	0.65 ±0.15	2755.41 ±225.34	0.76 ±0.08	3.21 ±0.24	nd
13 14, shoot	79.88 ±5.91	18.37 ±1.31	6.82 ±5.88	0.70 ±0.48	2871.92 ±219.99	0.78 ±0.09	3.68 ±0.14*	nd
WT, root	239.66 ±18.67	34.19 ±3.12	6.29 ±1.31	0.64 ±0.15	7621.02 ±652.67	0.95 ±0.04	9.87 ±0.42	nd
13 14, root	236.73 ±25.73	31.13 ±3.15	5.72 ±1.07	0.63 ±0.23	6744.39 ±1114.97	0.87 ±0.07	7.35 ±2.24	nd

Hormone levels (ng/g DW) are shown as mean values of triplicates ±SD.
nd, Not detected at quantifiable levels.
WT, wild type.
13 14, *sweet13 sweet14*.
*Significantly different compared to the values in wild type (P<0.05) by Student's t-test.
**Significantly different compared to the values in wild type (P<0.001) by Student's t-test.
IAA, indole acetic acid; ABA, abscisic acid; JA, jasmonic acid; JA-Ile, jamonoyl-isoleucine; SA, salicylic acid; iP, isopentenyladenine; tZ, *trans*-zeatin; DHZ, dihydrozeatin.

Supplementary Table 2. Extraction, purification, and LC separation of samples.

Sample type	Analysed hormones	Sample weight	Extraction solution	Volume of extraction solution (mL)	Purification column (bed volume)				LC methods (SupplementaryTable 3)
					Step 1	Step 2	Step 3	Step 4	
Flowers	14GAs	3-10 mg DW	80% acetone containing 1% acetic acid	2	HLB (1 cc)	DEA (1 cc)	SepPak silica (1 cc)	-	4
Anthers	14GAs	0.4-1.4 mg DW	80% acetone containing 1% acetic acid	2	HLB (1 cc)	DEA (1 cc)	SepPak silica (1 cc)	-	4
Filaments	14GAs	0.9-3.4 mg DW	80% acetone containing 1% acetic acid	2	HLB (1 cc)	DEA (1 cc)	SepPak silica (1 cc)	-	4
Shoots	other hormones	30-40 mg DW	80% acetonitrile containing 1% acetic acid	6	HLB (1 cc)	MCX (1 cc)	WAX (1 cc)	-	1, 2, 3
Shoots	14GAs	27-35 mg DW	80% acetone containing 1% acetic acid	5	HLB (3 cc)	DEA (1 cc)	SepPak silica (1 cc)	-	4
Roots	other hormones	7-16 mg DW	80% acetonitrile containing 1% acetic acid	4	HLB (1 cc)	MCX (1 cc)	WAX (1 cc)	-	1, 2, 3
Roots	14GAs	18-30 mg DW	80% acetone containing 1% acetic acid	5	HLB (3 cc)	DEA (1 cc)	SepPak silica (1 cc)	-	4
Developing seeds	14GAs, other hormones	10-16 mg DW	80% acetonitrile containing 1% acetic acid	2	HLB (1 cc)	MCX (1 cc)	WAX (1 cc)	SepPak silica (1 cc)	1, 2, 3, 4
GA_3 transport assay in whole plants	GA_3	2-4 mg DW	80% acetonitrile containing 1% acetic acid	1	WAX (1 cc)	-	-	-	4
Yeast cells	GA_1	n.d.	80% acetone containing 1% acetic acid	1	WAX (1 cc)	-	-	-	4
	GA_3	n.d.	80% acetone containing 1% acetic acid	1	WAX (1 cc)	-	-	-	4
	GA_4	n.d.	80% acetone containing 1% acetic acid	1	WAX (1 cc)	-	-	-	4
	11GAs	n.d.	80% acetone containing 1% acetic acid	1	WAX (1 cc)	SepPak silica (1 cc)	-	-	4
	IAA	n.d.	80% acetone containing 1% acetic acid	1	WAX (1 cc)	-	-	-	1
	ABA	n.d.	80% acetone containing 1% acetic acid	1	WAX (1 cc)	-	-	-	1
	JA	n.d.	80% acetone containing 1% acetic acid	1	WAX (1 cc)	-	-	-	1
	JA-Ile	n.d.	80% acetone containing 1% acetic acid	1	WAX (1 cc)	-	-	-	1

n.d., not determined
DW, dry weight
FW, fresh weight

Supplementary Table 3. Purification by solid phase columns.

Column	Steps	
HLB (Oasis HLB; Waters)	1	Wash the columns successively with a bed volume of acetonitrile and methanol
	2	Equilibrate the columns with a bed volume of water containing 1% (v/v) acetic acid
	3	Load onto the columns the dried samples that are dissolved in water containing 1% (v/v) acetic acid
	4	Wash the column with a bed volume of water containing 1% (v/v) acetic acid
	5	Elute hormones twice with a bed volume of 80% (v/v) acetonitrile containing 1% (v/v) acetic acid
MCX (Oasis MCX; Waters)	1	Wash the columns successively with a bed volume of acetonitrile and methanol, followed by a one-half bed volume of 0.1 M KOH
	2	Equilibrate the columns with a bed volume of water containing 1% (v/v) acetic acid
	3	Load onto the columns the samples that are dissolved in water containing 1% (v/v) acetic acid
	4	Wash the columns several times with a bed volume of water containing 1% (v/v) acetic acid
	5	Elute the acidic and neutral fractions containing GAs, ABA, IAA, JA, JA-Ile, and SA twice with a bed volume of 80 % (v/v) acetonitrile containing 1 % (v/v) acetic acid
	6	Wash the columns several times with a bed volume of water containing 5% (v/v) ammonia
	7	Elute basic fractions containing cytokinins twice with a bed volume of 60% (v/v) acetonitrile containing 5% (v/v) ammonia
	8	Use 5% of the eluate after step 5 for SA analysis
WAX (Oasis WAX; Waters)	1	Wash the columns successively with a bed volume of acetonitrile and methanol, followed by a one-half bed volume of 0.1 M HCl
	2	Equilibrate the columns with a bed volume of water containing 1% (v/v) acetic acid
	3	Load onto the columns the dried samples (acidic and neutral fractions obtained by MCX purification) that are dissolved in water containing 1% (v/v) acetic acid
	4	Wash the columns several times with a bed volume of water containing 1% (v/v) acetic acid and then with bed volume of acetonitrile
	5	Elute acidic fractions containing GAs, ABA, IAA, JA and JA-Ile twice with 80% (v/v) acetonitrile containing 1% (v/v) acetic acid
	6	Use 10 % of eluate after step 5 for the analysis of ABA, IAA, JA and JA Ile
DEA (Bound Elut DEA; Agilent)	1	Wash the columns with a bed volume of methanol
	2	Equilibrate the columns with a bed volume methanol
	3	Load onto the columns the dried samples that are dissolved in methanol
	4	Wash the columns several times with methanol
	5	Elute GAs twice with a bed volume of methanol containing 1% (v/v) acetic acid
SepPak silica (SepPak silica; Waters)	1	Wash and equilibrate the columns several times with a bed volume of chloroform:ethylacetate = 1:1 (v/v) containing % (v/v) acetic acid
	2	Load the dried samples that are dissolved in chloroform:ethylacetate = 1:1 (v/v) containing 1% (v/v) acetic acid
	3	Collect the flow-through containing GAs after step 2
	4	Further elute GAs twice with a bed volume of chloroform:ethylacate = 1:1 (v/v) containing 1% (v/v) acetic acid

Supplementary Table 4. Conditions of LC.

<table>
<tr><th>Method No.</th><th>Solvent A</th><th>Solvent B</th><th>Gradient (composition of solvent B)</th><th>column</th></tr>
<tr><td rowspan="5">1</td><td rowspan="5">Water containing 0.01% (v/v) acetic acid</td><td rowspan="5">MeCN containing 0.05% (v/v) acetic acid</td><td>Constant at 3% for 0.5 min</td><td rowspan="5">ZORBAX Eclipse XDB-C18 column (Agilent , 18 μm, 2.1 × 50 mm)</td></tr>
<tr><td>Linear gradient from 3 to 15% over 0.5 min</td></tr>
<tr><td>Constant at 15% for 2min</td></tr>
<tr><td>Linear gradient from 15 to 40% over 4 min</td></tr>
<tr><td>Linear gradient from 40 to 60% over 1 min</td></tr>
<tr><td rowspan="3">2</td><td rowspan="3">Water containing 0.01% (v/v) acetic acid</td><td rowspan="3">MeCN containing 0.05% (v/v) acetic acid</td><td>Constant at 3% for 0.5 min</td><td rowspan="3">ZORBAX Eclipse XDB-C18 column (Agilent, 18 μm, 2.1 × 50 mm)</td></tr>
<tr><td>Linear gradient from 3 to 10% over 2.5 min</td></tr>
<tr><td>Linear gradient from 10 to 40% over 2 min</td></tr>
<tr><td rowspan="2">3</td><td rowspan="2">Water containing 0.1% (v/v) formic acid</td><td rowspan="2">MeCN containing 0.1% (v/v) formic acid</td><td>Constant at 3% for 0.5 min</td><td rowspan="2">ACQUITY UPLC BEH C18 column (Waters, 17 μm, 2.1 × 50 mm)</td></tr>
<tr><td>Linear gradient from 3 to 97% over 7 min</td></tr>
<tr><td rowspan="4">4</td><td rowspan="4">Water containing 0.01% (v/v) acetic acid</td><td rowspan="4">MeCN containing 0.05% (v/v) acetic acid</td><td>Constant at 3% linear for 0.5 min</td><td rowspan="4">ACQUITY UPLC BEH phenyl column (Waters, 17 μm, 2.1 × 50 mm)</td></tr>
<tr><td>Linear gradient from 3 to 20% over 2.5 min</td></tr>
<tr><td>Linear gradient from 20 to 40% over 5 min</td></tr>
<tr><td>Constant at 40% for 2 min</td></tr>
</table>

Supplementary Table 5. Parameters of tandem mass spectrometer.

Compound	LC method	Retention time on LC (min)	Polarity of ESI	IonSpray voltage (kV)	Desolvation temperature (°C)	Declustering potential (V)	Collision energy (V)	Precursor ion (*m/z*)	Scan range (*m/z*)	Qualifier ion (*m/z*)
D_2-GA_1	1	2.8	-	-3.5	600	-90	-30	349.2	100-400	275.2
GA_1	1		-	-3.5	600	-90	-30	347.2	100-400	273.2
D_2-IAA	1	4.0	-	-3.5	600	-90	-15	176.1	100-200	132.1
IAA	1		-	-3.5	600	-90	-15	174.1	100-200	130.1
D_6-ABA	1	5.2	-	-3.5	600	-90	-15	269.1	100-300	159.1
ABA	1		-	-3.5	600	-90	-15	263.1	100-300	153.1
D_2-JA	1	6.1	-	-3.5	600	-90	-20	211.1	50-250	59.0
JA	1		-	-3.5	600	-90	-20	209.1	50-250	59.0
D_2-GA_4	1	7.1	-	-3.5	600	-90	-30	333.2	100-400	259.2
GA_4	1		-	-3.5	600	-90	-30	331.2	100-400	257.2
$^{13}C_6$-JA-Ile	1	7.4	-	-3.5	600	-90	-30	328.2	50-350	136.1
JA-Ile	1		-	-3.5	600	-90	-30	322.2	50-350	130.1
D_5-tZ	2	2.6	+	4.5	600	80	30	225.1	50-250	136.1/137.1
tZ	2		+	4.5	600	80	30	220.1	50-250	136.1
D_3-DHZ	2	2.7	+	4.5	600	80	30	225.1	50-250	136.1
DHZ	2		+	4.5	600	80	30	222.1	50-250	136.1
D_6-iP	2	4.5	+	4.5	600	80	30	210.1	50-250	137.1
iP	2		+	4.5	600	80	30	204.1	50-250	136.1
D_6-SA	3	3.1	-	-4.0	600	-10	-25	141.1	50-1000	97.1
SA	3		-	-4.0	600	-10	-25	137.1	50-1000	93.0
D_2-GA_8	4	2.3	-	-4.0	600	-90	-30	365.2	100-1000	277.2
GA_8	4		-	-4.0	600	-90	-30	363.2	100-1000	275.2
D_2-GA_{29}	4	2.5	-	-4.0	600	-90	-40	349.2	100-1000	305.2
GA_{29}	4		-	-4.0	600	-90	-40	347.2	100-1000	303.2
D_2-GA_1	4	3.3	-	-4.0	600	-90	-40	349.2	100-1000	275.2
GA_1	4		-	-4.0	600	-90	-40	347.2	100-1000	273.2
D_2-GA_3	4	3.3	-	-4.0	600	-90	-40	347.2	100-1000	223.2
GA_3	4		-	-4.0	600	-90	-40	345.2	100-1000	221.2
D_2-GA_{19}	4	4.6	-	-4.0	600	-80	-45	363.2	100-1000	275.2
GA_{19}	4		-	-4.0	600	-80	-45	361.2	100-1000	273.2
D_2-GA_{20}	4	4.8	-	-4.0	600	-90	-35	333.2	100-1000	289.2
GA_{20}	4		-	-4.0	600	-90	-35	331.2	100-1000	287.2
D_2-GA_{44}	4	5.0	-	-4.0	600	-90	-40	347.2	100-1000	303.2
GA_{44}	4		-	-4.0	600	-90	-40	345.2	100-1000	301.2
D_2-GA_{34}	4	5.5	-	-4.0	600	-90	-40	349.2	100-1000	261.2
GA_{34}	4		-	-4.0	600	-90	-40	347.2	100-1000	259.2
D_2-GA_{51}	4	5.6	-	-4.0	600	-90	-35	333.2	100-1000	289.2
GA_{51}	4		-	-4.0	600	-90	-35	331.2	100-1000	287.2
D_2-GA_{53}	4	5.7	-	-4.0	600	-90	-40	349.2	100-1000	305.2
GA_{53}	4		-	-4.0	600	-90	-40	347.2	100-1000	303.2
D_2-GA_4	4	6.6	-	-4.0	600	-90	-30	333.2	100-1000	259.2
GA_4	4		-	-4.0	600	-90	-30	331.2	100-1000	257.2
D_2-GA_{24}	4	6.8	-	-4.0	600	-90	-40	347.2	100-1000	259.2
GA_{24}	4		-	-4.0	600	-90	-40	345.2	100-1000	257.2
D_2-GA_{15}	4	8.0	-	-4.0	600	-90	-40	331.2	100-1000	259.2
GA_{15}	4		-	-4.0	600	-90	-40	329.2	100-1000	257.2
D_2-GA_9	4	8.0	-	-4.0	600	-90	-35	317.2	100-1000	273.2
GA_9	4		-	-4.0	600	-90	-35	315.2	100-1000	271.2
D_2-GA_{12}	4	8.7	-	-4.0	600	-90	-40	333.2	100-1000	315.2
GA_{12}	4		-	-4.0	600	-90	-40	331.2	100-1000	313.2

Supplementary References

1. Nakabayashi, K., Okamoto, M., Koshiba, T., Kamiya, Y. & Nambara, E. Genome-wide profiling of stored mRNA in *Arabidopsis thaliana* seed germination: epigenetic and genetic regulation of transcription in seed. *Plant J.* **41**, 697-709 (2005).

2. Schmid, M. et al. A gene expression map of *Arabidopsis thaliana* development. *Nat. Genet.* **37**, 501-506 (2005).

3. Winter, D. et al. An "Electronic Fluorescent Pictograph" browser for exploring and analyzing large-scale biological data sets. *PLOS ONE* **2**, e718 (2007).
